# Monitoring of Salinity, Temperature, and Drought Stress in Grafted Watermelon Seedlings Using Chlorophyll Fluorescence

**DOI:** 10.3389/fpls.2021.786309

**Published:** 2021-12-22

**Authors:** Yu Kyeong Shin, Shiva Ram Bhandari, Jun Gu Lee

**Affiliations:** ^1^Department of Horticulture, College of Agriculture & Life Sciences, Jeonbuk National University, Jeonju, South Korea; ^2^Core Research Institute of Intelligent Robots, Jeonbuk National University, Jeonju, South Korea; ^3^Institute of Agricultural Science & Technology, Jeonbuk National University, Jeonju, South Korea

**Keywords:** proline, abiotic stress, maximum quantum yield, growth parameter, chlorophyll

## Abstract

Chlorophyll fluorescence (*CF*) is used to measure the physiological status of plants affected by biotic and abiotic stresses. Therefore, we aimed to identify the changes in *CF* parameters in grafted watermelon seedlings exposed to salt, drought, and high and low temperatures. Grafted watermelon seedlings at the true three-leaf stage were subjected to salinity levels (0, 50, 100, 150, and 200 mM) and temperature [low (8°C), moderate (24°C), and high (40°C)] stresses for 12 days under controlled environmental conditions independently. Eight *CF* parameters were measured at 2-day intervals using the FluorCam machine quenching protocol of the FluorCam machine. The seedlings were also exposed to drought stress for 3 days independent of salinity and temperature stress; *CF* parameters were measured at 1-day intervals. In addition, growth parameters, proline, and chlorophyll content were evaluated in all three experiments. The *CF* parameters were differentially influenced depending on the type and extent of the stress conditions. The results showed a notable effect of salinity levels on *CF* parameters, predominantly in maximum quantum yield (Fv/Fm), non-photochemical quenching (NPQ), the ratio of the fluorescence decrease (Rfd), and quantum yield of non-regulated energy dissipation in PSII [Y(NO)]. High temperature had significant effects on Rfd and NPQ, whereas low temperature showed significant results in most *CF* parameters: Fv/Fm, Y(NO), NPQ, Rfd, the efficiency of excitation capture of open photosystem II (PSII) center (Fv′/Fm′), and effective quantum yield of photochemical energy conversion in PSII [Y(PSII)]. Only NPQ and Rfd were significantly influenced by severe drought stress. Approximately, all the growth parameters were significantly influenced by the stress level. Proline content increased with an increase in stress levels in all three experiments, whereas the chlorophyll (a and b) content either decreased or increased depending upon the stressor. The results provided here may be useful for understanding the effect of abiotic stresses on *CF* parameters and the selection of index *CF* parameters to detect abiotic stresses in grafted watermelon seedlings.

## Introduction

Plants experience a range of stresses during their life cycle and exhibit physiological, biochemical, and molecular responses to biotic and abiotic stresses (Fahad et al., 2017; Nadeem et al., 2018). These stressors affect the plants negatively in different ways, depending on the extent and duration of the stress (Shin et al., 2020a; Giordano et al., 2021). The ultimate effects of stressors are reduction in growth by decreasing the photosynthesis rate, changes in bioactive compounds, and overall yield (Toscano et al., 2019; Giordano et al., 2021). Among the different abiotic stresses, salt, temperature, and drought stress are some of the important abiotic stresses experienced by plants during cultivation (Kalaji et al., 2016). The effects of each abiotic stress slightly differ from each other to some extent, although their ultimate effects are the reduction in growth *via* reduced photosynthesis rate, alteration in phytochemicals, and overall yield. The effects of stressors in plants have been studied in a range of plants at different stages of their life cycle (Bhandari et al., 2018; Shin et al., 2020a,b).

Salinity stress disrupts membrane permeability and stomatal closure, and imbalances ion concentrations; this reduces the photosynthetic rate as well as the levels of photosynthetic pigments, growth, and yields by up to 20% worldwide (De Oliveira et al., 2013). Salinity stress influences the relationship between salinity level and water, stomatal closure, leaf wilting, premature aging of leaves due to salinity accumulation, and decreased growth and yield (Garg et al., 2020; Lotfi et al., 2020). Salinity stress can be due to either a short-term exposure or a long-term stress due to continuous nutrient and salinity accumulation in the rhizome, affecting growth and fruit production (Negrão et al., 2017). Plants may receive either high- or low-temperature stresses during cultivation; their effects are dependent on the plant genotypes (Korkmaz and Dufault, 2001; Hou et al., 2016; Rajametov et al., 2021). In particular, high-and low-temperature stress causes various physiological changes in plants, such as damage to the cellular structure of plants, reduction of chlorophyll levels, and deterioration of photosynthetic function (Garstka et al., 2007; Mattila et al., 2020). High temperatures severely affect the structure and functions of cell membranes, causing early bolting, dehydration of soil moisture content, and disruption of ion movement, which reduces photosynthesis and ~50% reduction in total yield in different crops (Fahad et al., 2017; Nadeem et al., 2018). Under high temperature stress, plants accumulate reactive oxygen species (ROS), such as hydrogen peroxide (Soengas et al., 2018; Hassan et al., 2020). Furthermore, the electron transport ability during photosynthesis is reduced, reducing the energy utilization capacity of photosystem II (Song et al., 2014). In contrast, excessively low temperatures are responsible for chilling injuries in plants and damage to the photosynthetic apparatus (Lee et al., 2021). Soil water content has a crucial role, and optimum water content is required for plant growth under normal conditions. A reduction in soil water content, causing drought stress in plants, disrupts the uptake of minerals and other essential nutrients (Yordanov et al., 2000). Sometimes, sudden changes in temperature levels during the life cycle of plants cause thermal shock which may induce the expression of some genes which in turn results in the increased synthesis of some proteins: heat shock proteins (Gupta et al., 2010). These proteins are responsible to bind and stabilize misfolded proteins. Such proteins are also induced by the other stress factors such as osmotic potential, salinity, drought, and high intensity irradiations (Swindell et al., 2007). Furthermore, plants also undergo various *in vitro* phenotypic and *in vivo* physiological changes both internally and externally when subjected to drought stress, and activate multiple defense mechanisms for protection and survival (Yordanov et al., 2000; Kapoor et al., 2020; Tabassum et al., 2021). In addition, drought stress is known to limit photosynthetic activity (Shafiq et al., 2021). Drought stress caused by inadequate watering induces physiological changes in plants, such as cell dehydration, osmotic pressure imbalance, and plant growth retardation (Jia et al., 2021). Depending on abiotic stressors, such as salinity, drought, high, and low temperatures, plants show various phenotypic and physiological responses *in vivo* according to various crops, genetic resources, varieties, growth stages, and stress tolerance levels (He et al., 2018). Photo-inhibition caused by environmental stress on photosystem II is closely related to the photosynthetic performance of plants (Murata et al., 2007). Therefore, studying the effects of abiotic stress in plants is an important step in the production of high-yield and nutritionally improved crops (Fahad et al., 2017; Liang et al., 2020).

Both destructive and non-destructive methods have been used to detect abiotic stress and their responses in plants (Gorbe and Calatayud, 2012; Kalaji et al., 2016; Bhandari et al., 2018; Susič et al., 2018; Shin et al., 2020b). Among them, chlorophyll fluorescence (*CF*) imaging is one of the most common non-destructive techniques that has been applied to detect abiotic stresses in a range of plants (Moustakas et al., 2021). The *CF* parameters provide information on the mechanical detail and extent of damage in plants due to stress. Protocols capable of measuring various chlorophyll fluorescence parameters include the chlorophyll fluorescence induction curve (OJIP), the Kautsky effect (chlorophyll a fluorescence induction), and quenching effects (Lichtenthaler and Babani, 2000; Zushi et al., 2012; Yao et al., 2018; Shin et al., 2020a).

Watermelon (*Citrullus lanatus*) is a high-income economic crop, with 3.08 million hectares of cultivated area and 100 million tons of worldwide production (FAO, 2019). It has been found that the overall yield and fruit quality of watermelon are severely affected by biological and environmental stressors during cultivation (Toscano et al., 2019; Giordano et al., 2021). To solve this problem, the development of particular stress-resistant cultivars has been pursued; however, it requires diverse strategies, sufficient time, and human resources (Bulgari et al., 2019). As an alternative, the use of stress-resistant stock cultivars and grafted seedlings for high quality and yield is steadily increasing (Kumar et al., 2017). As the use of grafted seedlings has increased in response to various stressors, the domestic seedling market is developing into specialized seedling production facilities with expertise in facility gardening, smart farms, and grafting machines (Kwack et al., 2021). The development of the seedling industry reflects various cultivation management objectives, such as of soil-borne diseases in the growing environment, climate change, and increases in fruit production (Rahmatian et al., 2014; Spanò et al., 2020). Furthermore, seedling industries have been used small container (plug tray having different sizes) for the efficient production of seedlings as it occupies small space and cost effective. The various abiotic factors affecting grafted seedlings include lack of nutrients, salinity accumulation, drought, water, and high and low temperatures (Coskun et al., 2016; Nievola et al., 2017; Hussain et al., 2018). Both grafted and non-grafted seedlings have been used for watermelon cultivation; however, the use of grafted seedlings has been increasing because of their high yield (Lee et al., 2010). Several studies related to the effects of abiotic stress in watermelon have been performed (Yetişir and Uygur, 2009; Hou et al., 2016; Yanyan et al., 2018; Lu et al., 2020, 2021). However, the effects of salinity, temperature, and drought stresses independently in a single cultivar during a different treatment schedule have not been performed in detail.

Therefore, this study was performed to evaluate the effects of salt, temperature, and drought stress on *CF* parameters, photosynthetic pigments (chlorophyll a and b), stress-related compounds (proline), and growth performance in grafted watermelon seedlings during progressive exposure to the respective stressors, and to select possible index *CF* parameters for the detection of salt, temperature, and drought stress.

## Materials and Methods

### Plant Material and Seedling Preparation

For the preparation of watermelon (*Citrullus lanatus*) grafted seedlings, the scion cultivar ‘Seo Tae Ja’ (Asia Seed Co. Ltd., Seoul, South Korea) and stock cultivar ‘Seol Jung Mae Plus’ (Tae Seong Seed Co. Ltd., Yeonggwang, South Korea) which was resistant to temperature stress were used. The scion seeds were sowed in 128-cell plug trays (54.4 cm × 28.2 cm × 5.4 cm); stock seeds were sowed in 40-cell plug trays (54.4 cm × 28.2 cm × 5.4 cm) filled with bed soil (Chorok-i, Nongwoobio Co. Ltd., Suwon, South Korea). Grafted seedlings were made from the scion and stock using the single cotyledon grafting method, according to Hassell et al. (2008), and were used in experiments when the true leaves of the scion reached the three-leaf stage. Watermelon-grafted seedlings were grown by a professional seedling company (Sol-Rae Seedling Farm, Iksan, South Korea) in a greenhouse with the standard protocol developed for experimental plant materials until the three true-leaf stages.

### Experimental Design and Growth Conditions

**Figure 1:**
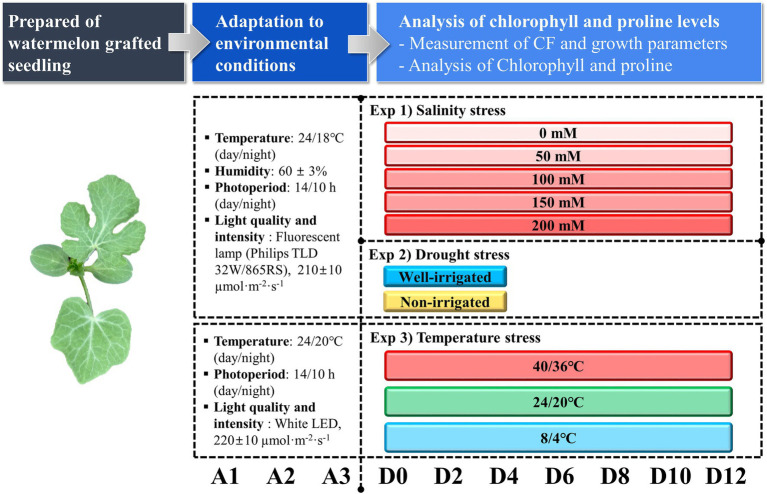
Overview of individual experimental processes and measurement timeline. A1, A2, and A3 represent the respective acclimatization times. D0–D12 represents the days after the treatment time.

The experiment was performed under three different stress conditions: salt, temperature, and drought stress independently. A detailed experimental plan is presented in Figure 1. For the salt and temperature stress treatments, the grafted seedlings were grown in a closed light box (120 cm × 65 cm × 45 cm; l × b × h) under a fluorescent lamp (Philips, TLD 32 W/865RS) with a photosynthetic photon flux density (PPFD) of 210 ± 10 μmol m^−2^ s^−1^, 24/18°C (day/night) temperature, 14/10-h (day/night) photoperiod, and 60 ± 3% relative humidity for 3 days of acclimatization. In the salt stress treatment, 35 seedlings were used for each treatment. The seedlings were treated with five different NaCl concentrations (0, 50, 100, 150, and 200 mM). Three liters of the respective NaCl solution were kept in different trays before irrigation, and the seedlings were irrigated once a day for 10 min in the morning until the end of the experiment. The seedlings were grown under the same conditions for 12 days. Drought stress treatment followed the same conditions for acclimatization. For the drought stress experiment, one set of grafted seedlings was irrigated every day with the nutrient solution EC 1.5 dS^−1^ using the sub-irrigation method for 20 min as the control (well-irrigated), whereas another set of seedlings was not irrigated (non-irrigation) after the start of the experiment for 3 days and assumed to be drought stressed. The conditions of the closed light box were similar to those of the salt stress experiment. The drought stress treatment was performed only for 3 days as the seedlings under drought stress showed permanent wilting symptoms with severe leaf deformation and stunted plant growth. In the temperature stress treatment, the seedlings were grown under the same light conditions, 24/20°C (day/night) temperature, 14/10-h (day/night) photoperiod, and non-controlled relative humidity for 3 days for acclimatization of the seedlings. The seedlings were then grown under three temperature conditions: low [8/4°C (day/night)], moderate [24/20°C (day/night)], and high [40/36°C (day/night)] for 12 days at the same light conditions. Irrigation was performed daily in the morning using the sub-irrigation method. Three liters of water was kept in each tray and the plug tray was embedded for 20 min in every morning and transferred to the respective temperature controller system.

### Measurement of Chlorophyll Fluorescence Parameters

An open FluorCam 800 (Photon System Instruments, Drasow, Czech Republic) was used for imaging of *CF* kinetics from the upper surface of all true leaves from the intact seedlings according to Shin et al. (2020b). Cool white 6,500 K in the LED panels (130 mm × 130 mm) was used as the light source at an angle of 45°. Seedlings were adapted in dark condition for 20 min before the measurement of *CF* parameters. The distance between the camera lens and the seedling canopy was maintained at 15–20 cm. In summary, eight *CF* parameters were assessed according to Shin et al. (2020b) using the following protocols: quenching act 2, shutter speed 20 μs, sensitivity 20%, actinic light 240 μmol m^−2^ s^−1^, and the saturating flash light 300 μmol m^−2^ s^−1^. Detailed information on each *CF* parameter is provided in Table 1. For the salinity and temperature stress treatments, seven watermelon-grafted seedlings (from 35 seedlings per treatment) were randomly selected for each time point (0, 2, 4, 6, 8, 10, and 12 days after the initiation of salinity stress) and used to measure the *CF* parameters. In contrast, the *CF* parameters were measured every day until day 3 of the drought stress initiation. Five seedlings (from 20 seedlings per treatment) were randomly selected for each measurement time (0, 1, 2, and 3 days) and used for the analysis of *CF* parameters.

**Table 1:** Chlorophyll fluorescence parameters used in this study.

Parameter	Formula	Description
Fv/Fm	(Fm − F0)/Fm	Maximum quantum yield of PSII photochemistry measured in the dark-adapted state
Fv′/Fm′	(Fm′ − F0’)/Fm′	Exciton transfer efficiency from antenna pigments to the reaction center of photosystem II (PSII) in the light-adapted state
Y(PSII)	(Fm′ − Fs)/Fm′	Effective quantum yield of photochemical energy conversion in PSII
NPQ	(Fm − Fm′)/Fm′	Non-photochemical quenching of maximum fluorescence
qP	(Fm′ − Fs)/(Fm′ − F′0)	Photochemical quenching of PSII
qN	(Fm − F’m)/(Fm − F′0)	Coefficient of non-photochemical quenching of variable fluorescence
Y(NO)	1/[NPQ + 1 + qL(Fm/F0–1)]	Quantum yield of non-regulated energy dissipation in PSII
Rfd	(Fm − Fs)/Fs	Ratio of fluorescence decline

### Measurement of Growth Parameters and Soil Moisture Content

Growth parameters included the number of leaves, shoot fresh and dry weights of the scion, plant height of scion and stock, and root fresh weight. Plant height was measured using a set of digital calipers (CD-20APX; Mitutoyo Co., Kanagawa, Japan) to evaluate the growth performance of the grafted seedlings. Fresh shoot and root weights were measured using a digital weighing machine (UX420H; Shimadzu Corp., Kyoto, Japan). The relative water content of the soil in a single plug tray was measured by drying the soil samples at 105°C for 72 h according to Shin et al. (2021a). The seedlings from 0, 6, and 12 of treatment time after measuring *CF* and growth parameters were collected separately, freeze-dried and used for chlorophyll and proline analysis in salinity and temperature stress treatments, whereas the samples from all the treatment times (0, 1, 2, and 3 days after the initiation of treatment) were used in the drought stress experiment. Seven seedlings from each treatment and time were mixed independently, freeze-dried, ground into a fine powder, and stored at −20°C for the analysis of chlorophyll and proline content.

### Analysis of Chlorophyll and Proline Content

Chlorophyll a and b were measured according to Shin et al. (2020b) using a microplate reader (Multiskan Go; Thermo Scientific Inc.). Twenty milligrams of freeze-dried and finely powdered samples were mixed with 10 ml MeOH (Avantor Performance Materials Co., Center Valley, PA, United States) for 2 h at room temperature (~25°C), and the aliquot was centrifuged at 2,400 × *g* for 10 min. The supernatant was filtered using a 0.45 μm syringe filter, and the absorbance was measured at 652 and 665 nm using a microplate reader.

The method for analysis of proline content was adopted from Shin et al. (2020b). A total of 50 mg of freeze-dried and powdered samples was mixed in 5 ml of 3% aqueous sulfosalicyclic acid dehydrate (Sigma-Aldrich, St. Louis, MO, United States), extracted for 1 h by shaking at 200 rpm, centrifuged (2,400 × *g* for 10 min), and filtered. The supernatant of the sample (500 μl), acetic acid from Sigma-Aldrich (500 μl), and acid ninhydrin from Sigma-Aldrich (500 μl) were mixed simultaneously in a 15-ml tube, kept in a water bath (at 95°C) for 1 h, and cooled rapidly on ice for 10 min. After adding 1 ml of toluene (Sigma-Aldrich) to the supernatant, the mixture was vortexed and centrifuged at 2,400 × *g* for 10 min. Thereafter, the toluene phase (200 μl) was added in a 96-well plate, and the absorbance was measured using a microplate reader at 520 nm. Proline content was quantified using a commercial L-proline (Sigma-Aldrich) standard with a linear range of 0–100 μg ml^−1^.

### Statistical Analyses

The results of growth parameters and *CF* parameters in salinity and temperature stress treatments are reported as the mean of seven biological replications, whereas the results were reported as the mean of five biological replications in drought stress treatment. The chlorophyll and proline contents were reported as the mean of three replicates in all the three experiments. Statistical analyses were performed using RStudio ver. 4.0.2 (R Studio Desktop, Boston, MA, United States). Statistical analysis followed by Duncan’s multiple range test was used to analyze the statistical differences among the mean values at *p* < 0.05. The interactive effect of respective treatments and treatment time were analyzed using a mixed model of one-way analysis of variance.

## Results

### Effect of Salinity Level on Growth and *CF* Parameters

**Figure 2:**
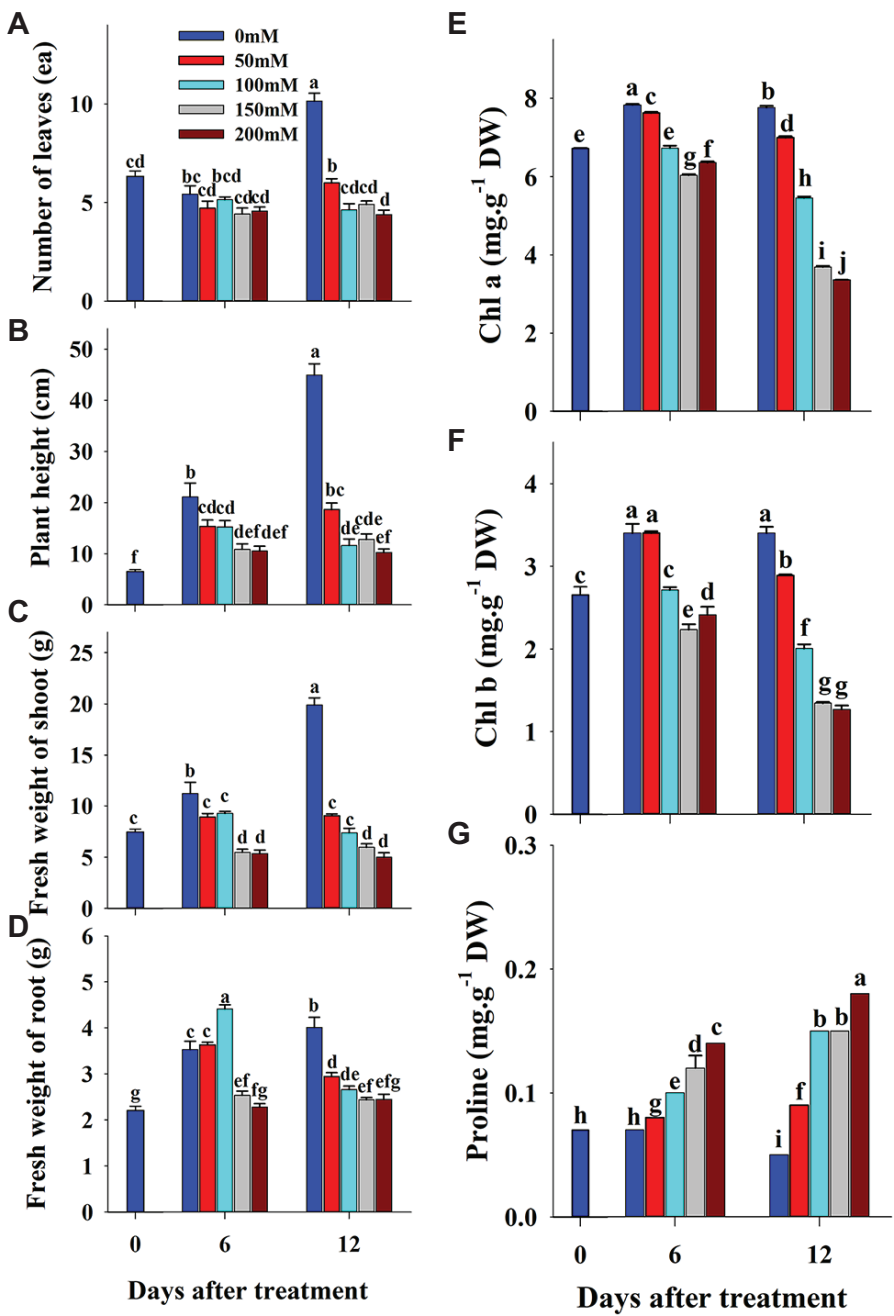
Changes in growth parameters **(A–D)**, chlorophyll **(E,F)** and proline **(G)** content of grafted watermelon seedlings grown under salinity stress during the progressive treatment time. Each bar represents the mean ± SD of seven biological replicates in growth parameters, and three replicates in chlorophyll and proline content. Different letters within a figure indicate a significant difference at *p* < 0.05 by Duncan’s multiple range test.

**Figure 3:**
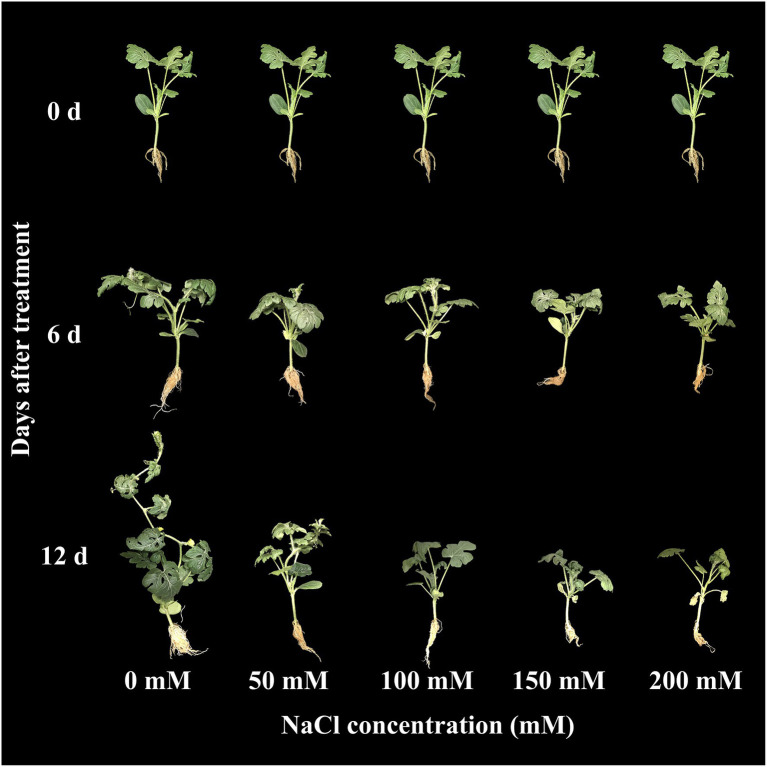
Changes in visual appearance of grafted watermelon seedlings grown under different salinity levels during the progressive treatment time.

The effect of salinity level on growth parameters (number of leaves, plant height, shoot fresh weight, and root fresh weight) is presented in Figures 2A–D. The growth parameters were measured at 0, 6, and 12 days of the experiment. The visual appearance was poor at salinity levels of 150 and 200 mM (Figure 3). Growth parameters gradually decreased with increasing salinity levels during the progressive treatment. The effect of salinity level was highest at 12 days after treatment initiation. The number of leaves significantly decreased at all salinity levels compared to the control at day 12 of treatment. Plant height, shoot fresh weight, and root fresh weight were also significantly lower in the seedlings grown under salinity stress than in the control. The effect of salinity level on plant height was relatively higher than on other growth parameters.

**Figure 4:**
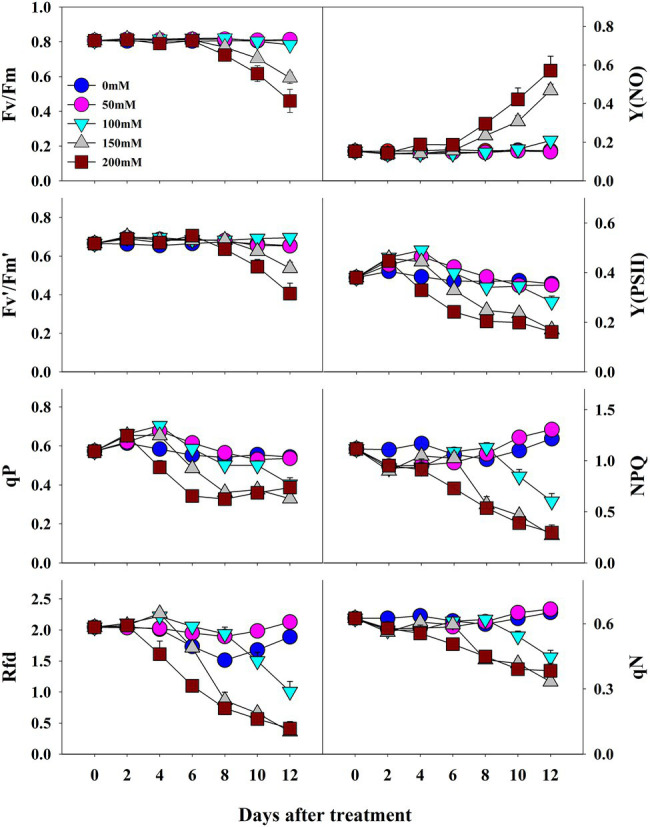
Changes in *CF* parameters in grafted watermelon seedlings grown under different salt concentration levels during the progressive treatment time. Each plot point represents the mean ± SD of seven biological replicates. Refer Table 1 for the description of each parameter.

The photochemical and non-photochemical quenching *CF* parameters measured at 2-day intervals were affected by salinity levels during the progressive treatment time (Figure 4). Except for Y(NO), all the other *CF* parameters decreased at higher salinity levels during the progressive treatment time. Fv/Fm, maximum quantum yield of PSII, showed non-significant changes until the end of the experiment (12 days of treatment time) at 0-, 50-, and 100-mM salinity levels. However, it decreased significantly at 150- and 200-mM salinity level from 8 day of treatment. Fv′/Fm′ also decreased at higher salinity levels in the later stage of treatment time; however, the magnitude of the decrease was lower than that of Fv/Fm. NPQ, an important non-photochemical quenching parameter, was not affected at the 50-mM salinity level throughout the experiment. It showed a gradual decrease from 6, 8, and 10 days after treatment at salinity levels of 200, 150, and 100 mM, respectively. Y(NO), a component that indicates the effectiveness of the photo-protection mechanism, started to increase from 8 days after treatment only at higher salinity levels (150 and 200 mM). Rfd also showed a similar changing pattern as in the NPQ at the respective salinity levels. qP, qN, and Y(PSII) also decreased with an increase in salinity level (>50 mM) from the 4 day of treatment. In summary, the results showed significant differences in all *CF* parameters according to salinity level, treatment duration, and their interactive effects (Table 2).

**Table 2:** Summary of analysis of *CF* parameters of watermelon grafted seedlings at five salinity levels (0, 50, 100, 150, and 200 mM) and seven treatment times (0, 2, 4, 6, 8, 10, and 12 days).

Parameters	Salinity level (S)		Treatment time (T)		S × T	
	F-Value	Significance	F-Value	Significance	F-Value	Significance
Fv/Fm	33.83	***	23.45	***	9.04	***
Fv′/Fm′	16.107	***	19.26	***	6.84	***
Y(PSII)	63.04	***	68.74	***	7.37	***
NPQ	80.57	***	15.70	***	15.62	***
qN	62.88	***	15.09	***	12.10	***
qP	41.987	***	46.18	***	6.05	***
Rfd	73.12	***	56.85	***	12.99	***
Y(NO)	46.05	***	30.95	***	10.03	***

*** indicate significance at *p* < 0.001. Detailed information on each CF parameter is provided in Table 1.

### Effect of Temperature Stress on Growth and Chlorophyll Fluorescence Parameters

**Figure 5:**
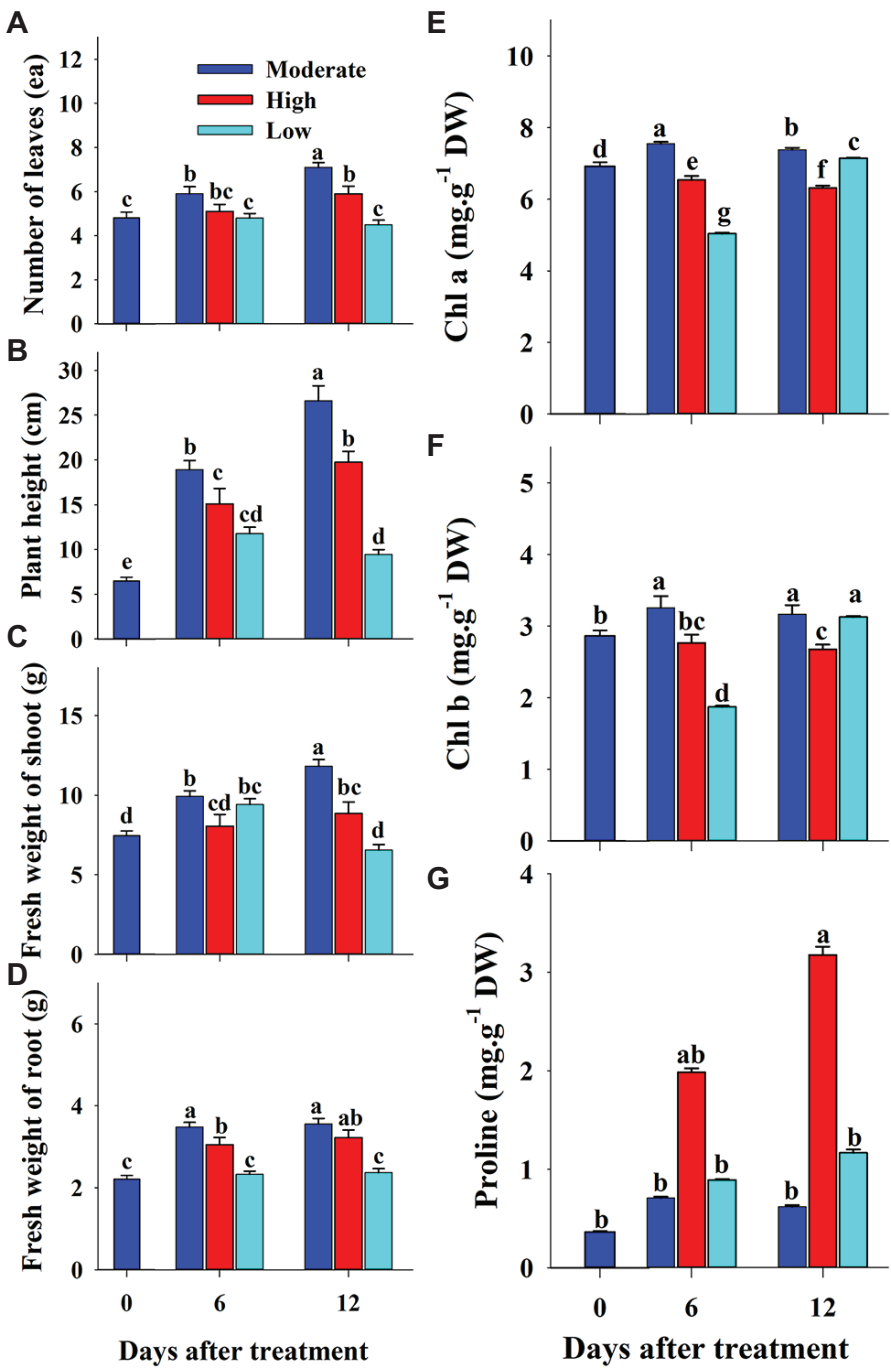
Changes in growth parameters **(A–D)**, chlorophyll **(E,F)** and proline **(G)** content of grafted watermelon seedlings grown under moderate, high, and low temperatures during the progressive treatment time. Each bar represents the mean ± SD of seven biological replicates in growth parameters, and three replicates in chlorophyll and proline content. Different letters within a figure indicate a significant difference at *p* < 0.05 by Duncan’s multiple range test.

**Figure 6:**
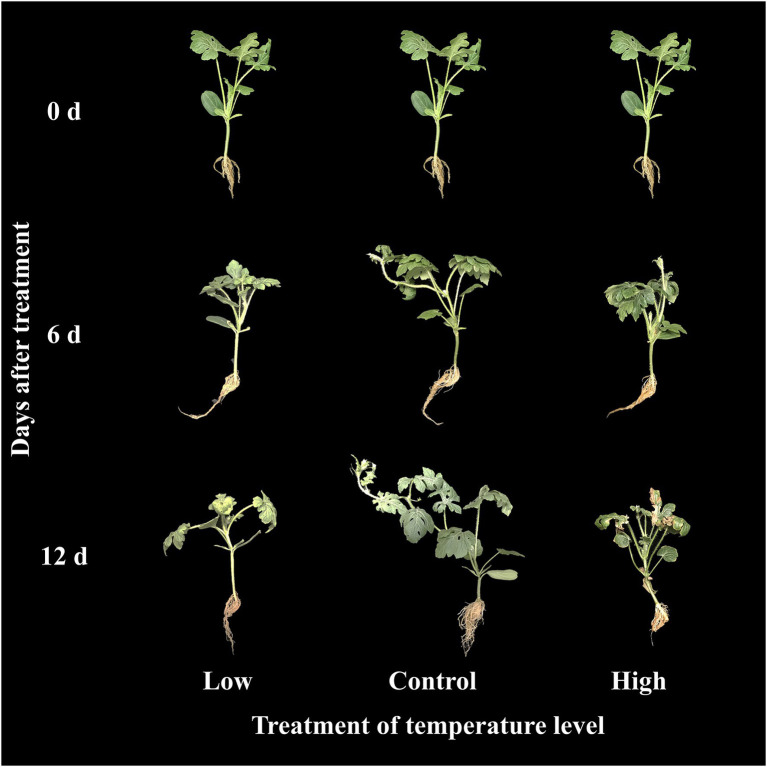
Changes in visual appearance of grafted watermelon seedlings grown under different temperature conditions during the progressive treatment time.

The effects of low, moderate, and high temperature on growth parameters at 0, 6, and 12 days of treatment are presented in Figure 5. All the growth parameters were lower in both the high and low temperature conditions compared to the control in respective treatment times. The total number of leaves and root fresh weight were statistically higher at the end of the experiment than in the beginning in both moderate and high temperature conditions; however, it remained constant throughout the experimental time at low temperature. Plant height and shoot fresh weight increased under moderate and high temperature conditions, whereas they increased at 6 days of treatment time and decreased with further treatment time at low temperature. Among the three treatment conditions, low temperatures showed significantly lower values of growth parameters at the end of the experiment. Although the growth parameters were highly reduced in the low-temperature treatment, the visual appearance of seedlings was as good as that in seedlings at moderate temperature (Figure 6). The leaves of seedlings grown under higher temperatures showed yellow burning leaves.

**Figure 7:**
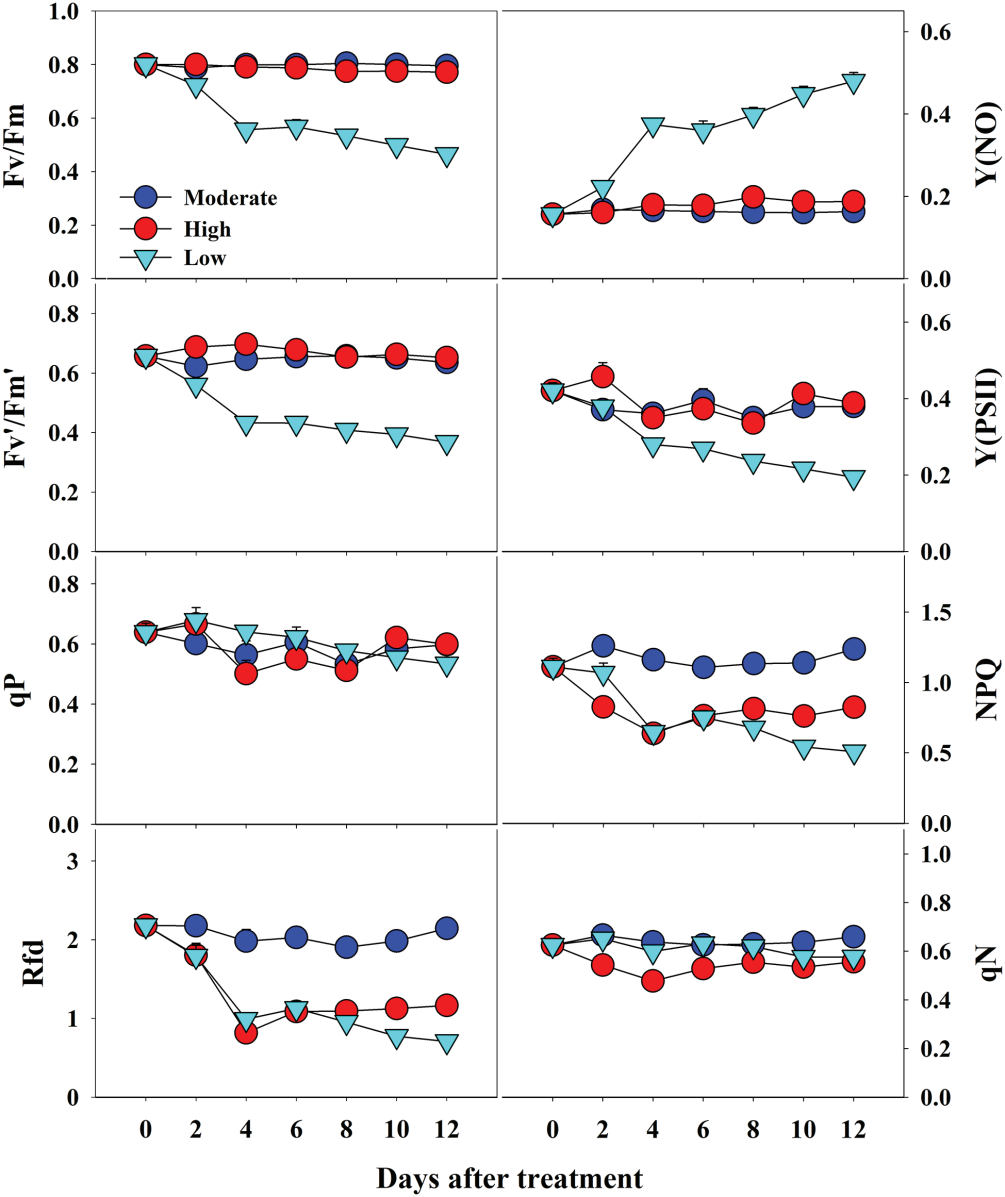
Changes in *CF* parameters in grafted watermelon seedlings grown under moderate, high and low temperature during progressive treatment time. Each plot point represents the mean ± SD of seven biological replicates. Refer Table 1 for the description of each parameter.

The *CF* parameters were differentially affected by the temperature conditions. Among the three temperature conditions, low temperature showed the highest effect on all *CF* parameters (Figure 7). Fv/Fm values continuously decreased from 0.81 at 0 day to 0.45 at the end of the experiment under low temperature treatment; they remained steady throughout the treatment time in high- and moderate-temperature conditions. A similar decreasing pattern was also observed in Fv′/Fm′ and Y(PSII) values in the seedlings at low temperatures. Rfd and NPQ were significantly affected by both high and low temperatures, showing a gradual decrease during progressive treatment time. In contrast, qP and qN showed non-significant changes under all temperature conditions. Only low temperature had a significant effect on Y(NO) levels, which increased gradually from the beginning and were highest at the end of the experiment. Overall, the effect of temperature stress, treatment time, and their interaction showed significant results for nearly all the *CF* parameters except for qP (Table 3).

**Table 3:** Summary of analysis of *CF* parameter of watermelon grafted seedlings at three temperature levels (8/4°C, 24/20°C, and 40/36°C day/night temperature) and seven treatment times (0, 2, 4, 6, 8, 10, and 12 days).

Parameters	Temperature level (T)		Treatment time (T)		T × T	
	F-Value	Significance	F-Value	Significance	F-Value	Significance
Fv/Fm	310.56	***	13.81	***	11.71	***
Fv′/Fm′	393.25	***	12.27	***	9.39	***
Y(PSII)	28.54	***	5.57	***	2.47	*
NPQ	83.63	***	7.37	***	5.98	***
qN	66.59	***	5.08	***	4.56	***
qP	2.41	NS	2.72	*	1.46	NS
Rfd	77.74	***	11.73	***	3.94	***
Y(NO)	253.10	***	14.75	***	11.84	***

*, and ***indicate significance at *p* < 0.05, and *p* < 0.001, respectively. NS: non-significant. Detailed information on each *CF* parameter is provided in Table 1.

### Effect of Drought Stress on Growth and Chlorophyll Fluorescence Parameters

**Figure 8:**
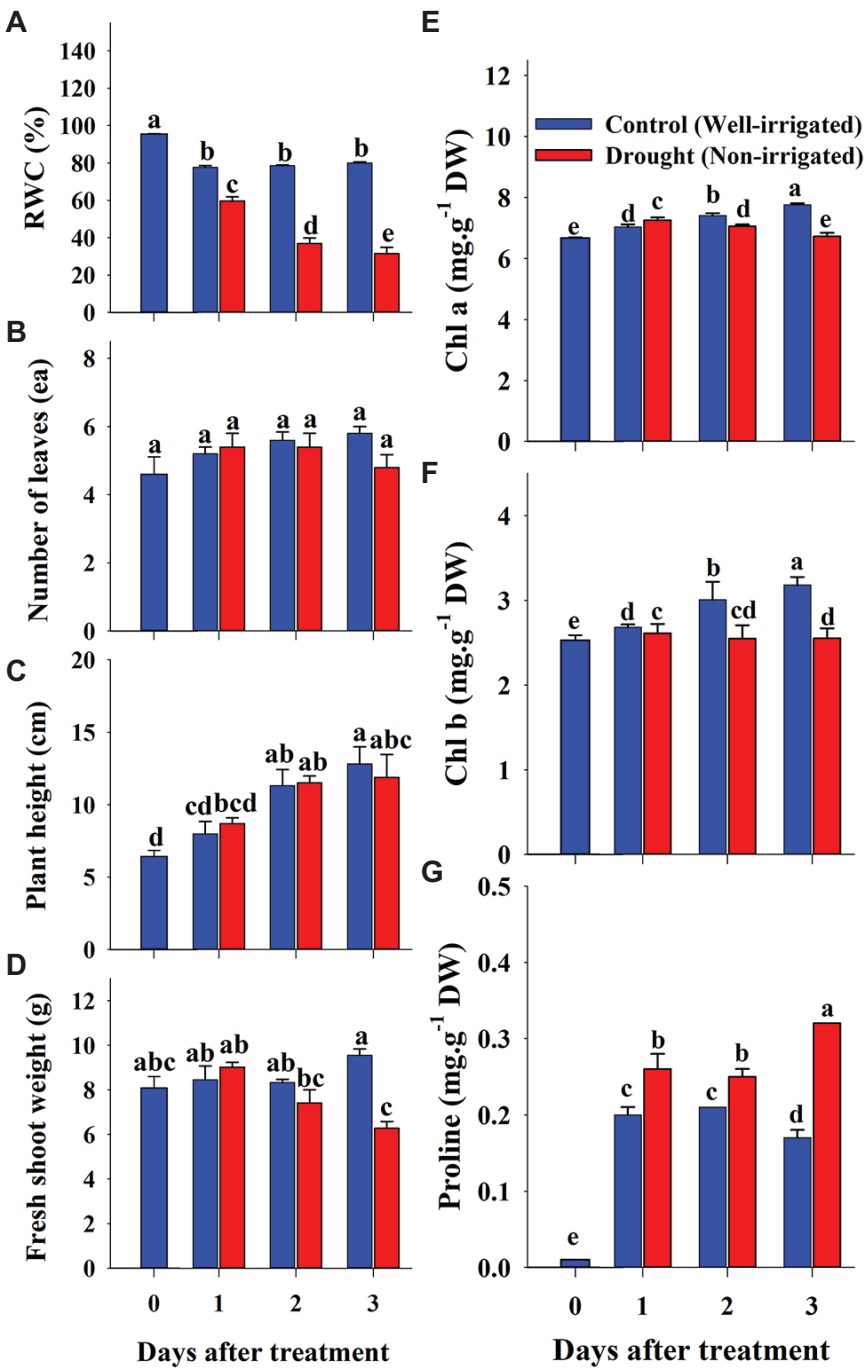
Changes in growth parameters **(A–D)**, chlorophyll **(E,F)** and proline **(G)** content of grafted watermelon seedlings grown under drought stress during the progressive treatment time. Each bar represents the mean ± SD of five biological replicates in growth parameters, and three replicates in chlorophyll and proline content. Different letters within a figure indicate a significant difference at *p* < 0.05 by Duncan’s multiple range test. RWC: relative water content.

**Figure 9:**
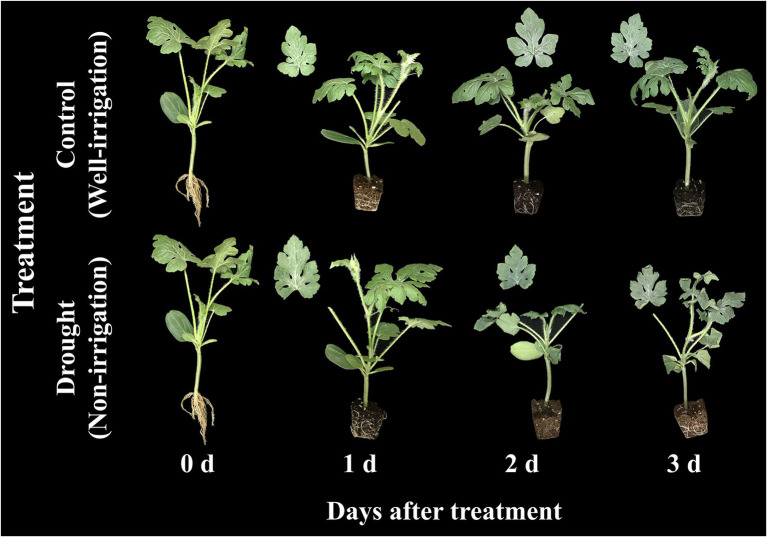
Changes in visual appearance of grafted watermelon seedlings grown under control and drought stress conditions during the progressive treatment time.

The changes in soil water content and growth parameters of watermelon seedlings during the progressive treatment time (0–3 days after treatment) under control (well-irrigation) and drought-stress (no-irrigation) treatments are presented in Figure 8. The water content in the soil gradually decreased from 77% (1^st^ day of treatment) to 37% (3^rd^ day of treatment time) under drought stress. The shoot fresh weight and number of leaves were statistically lower in drought-stressed seedlings than in control seedlings on the 3^rd^ day of the experiment. In contrast, plant height showed statistically similar values between control and drought stress groups at the respective treatment times, although the value was lower in drought-stressed seedlings than in the control at the end of the experiment. The shoot of the scion began to wither continuously after the initiation of drought stress, and the stock began to wilt on the 2^nd^ day (Figure 9). On the 3^rd^ day of drought stress, the seedlings had permanent wilting symptoms showing cotyledons and curling of first three true leaves downward and upward, respectively; therefore, further treatment was not performed.

**Figure 10:**
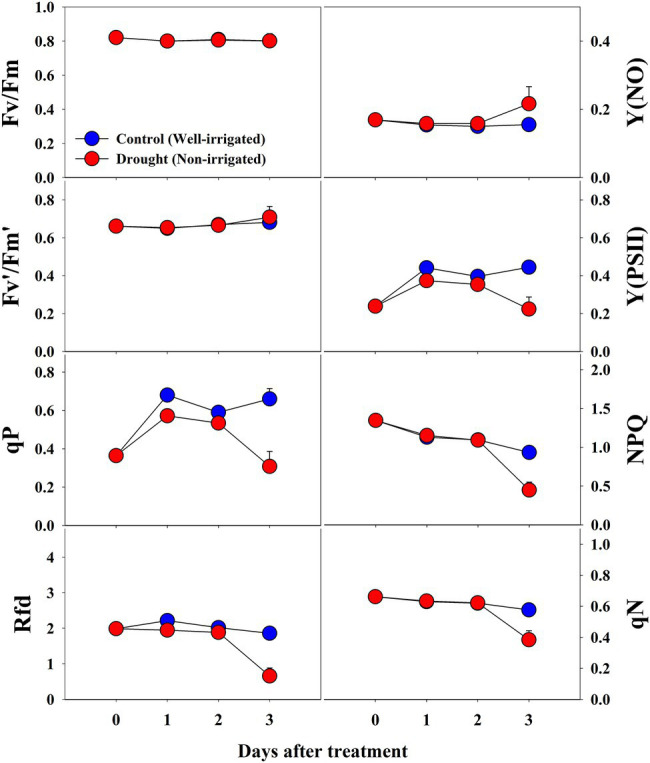
Changes in *CF* parameters in grafted watermelon seedlings grown under control and drought stress conditions during the progressive treatment time. Each plot point represents the mean ± SD of seven biological replicates. Refer to Table 1 for the description of each parameter.

Most of the *CF* parameters did not differ significantly between control and drought-stressed seedlings during the respective treatment times (Figure 10). Fv/Fm remained unchanged throughout the experiment between control and drought stress treatment, while Fv′/Fm′, Y(NO), qN, and NPQ showed significant changes only at the end of the experiment (3^rd^ day of treatment time). In contrast, Y(PSII), qP, and Rfd in drought stressed seedlings showed some decrement when compared to control seedlings at respective treatment time showing the statistically lower value at the end of the treatment time. At overall, except for Fv/Fm, all *CF* parameters were significantly affected by drought stress, treatment time, and their interaction (Table 4).

**Table 4:** Summary of analysis of *CF* parameters of watermelon grafted seedlings at two water levels (well-irrigated and non-irrigated) and four treatment times (0, 1, 2, and 3 days).

Parameters	Water level (W)		Treatment time (T)		W × T	
	F-Value	Significance	F-Value	Significance	F-Value	Significance
Fv/Fm	2.93	NS	1.11	NS	0.82	NS
Fv′/Fm′	8.25	**	17.58	***	6.51	**
Y(PSII)	89.41	***	3.82	*	21.22	***
NPQ	15.16	***	32.80	***	17.95	***
qN	39.51	***	57.78	***	41.52	***
qP	86.86	***	6.30	**	22.35	***
Rfd	68.04	***	26.91	***	23.86	***
Y(NO)	39.22	***	15.50	***	22.35	***

*, **, and *** indicate significance at *p* < 0.05, *p* < 0.01, and *p* < 0.001, respectively. NS: non-significant. Detailed information on each *CF* parameter is provided in Table 1.

### Effect of Salinity, Temperature, and Drought Stress on Chlorophyll and Proline Content

Chlorophyll and proline content were differentially affected by salinity, drought, and temperature stress. Content levels of both chlorophyll a and b decreased with increasing salinity levels and treatment time (Figures 2E,F). Chlorophyll a was decreased significantly with the increase of salinity level until 150 mM, and increased at 200 mM salinity level at 6 days of treatment time, while it continuously decreased with the increase of salinity level at 12 days of treatment time. Chlorophyll b followed somewhat similar pattern as shown in chlorophyll content at 6 days of treatment time while it was statistically similar in higher concentration (150 and 200 mM) at 12 days of treatment time. Chlorophyll b was more sensitive to salinity levels than chlorophyll a. It showed 29 and 63% of decrement in 6 and 12 days of treatment time, respectively in 200 mM salinity level compared to the control. Temperature stress also had a significant effect on both chlorophyll a and b content. Seedlings exposed to both high and low temperature conditions had statistically lower chlorophyll content levels at both 6 and 12 days of treatment compared to the control (Figures 5E,F). However, they were differentially affected by high and low temperatures. High temperature showed a continuous decrease in levels of both chlorophylls as the treatment time progressed, whereas both chlorophylls content decreased at day 6 and was restored at day 12 of low temperature stress. The effect of high temperature stress on the chlorophyll content was lower than that of the salinity stress treatment showing only 13 and 14% of decrement in 6 and 12 days of treatment time, respectively. Levels of both chlorophyll a and b increased at day 12 of treatment at low temperatures when compared with those at high temperatures. Seedlings under drought stress showed significantly higher chlorophyll a content levels than the control at the 1^st^ day and decreased from the 2^nd^ day on, whereas chlorophyll b levels were lower in drought-stressed seedlings than in the control from the 1^st^ day of treatment to the end of the experiment (Figures 8E,F).

The level of proline, an important stress indicator, increased with increases in salinity level at both 6 and 12 days of treatment time (Figure 2G), and the highest proline levels were found in the 200 mM-salinity level-treatment seedlings. Similarly, temperature stress also caused a significant change in proline content (Figure 5G). Plants subjected to both the high and low temperature treatment had higher proline content compared to the control at both the 6 and 12 day of treatment time (Figure 8G). High temperature stress showed the highest proline accumulation in both the 6 and 12 day of treatment time. Drought stress also resulted in higher proline content in stressed seedlings than in the control at different treatment times. Overall, the effect of each stressor, treatment time, and their interaction had significant effects on both chlorophyll and proline content (Table 5). Temperature stress showed the most significant changes (*F*-value: 5387; *p* > 0.001) in proline content within the stress levels of respective experiments, while both the chlorophyll a and b were highly affected by salinity stress than by the water and temperature stress.

**Table 5:** Summary of analysis of chlorophyll and proline of watermelon grafted seedlings at various salinity, drought, and temperature levels, and multiple treatment times.

Treatment	Parameter	Stress level (S)		Measurement treatment time (T)		S × T	
		F-Value	Significance	F-Value	Significance	F-Value	Significance
Salt	Chl a	9,147	***	7,891	***	1875	***
	Chl b	697.41	***	492.61	***	64.66	***
	Proline	963.06	***	62.02	***	11.93	***
Drought	Chl a	36.09	***	68.58	***	98.83	***
	Chl b	12.13	**	45.45	***	37.76	***
	Proline	889.36	***	285.41	***	61.56	***
Temperature	Chl a	572	***	203.4	***	532	***
	Chl b	73.41	***	44.46	***	97.33	***
	Proline	5387.5	***	399.7	***	469.2	***

** and *** indicate significance at *p* < 0.01 and *p* < 0.001, respectively. Chl: chlorophyll.

## Discussion

### Effect of Salt, Temperature, and Drought Stress on Growth Parameters

Plants experience many biotic and abiotic stresses during their life cycle. Salt, temperature, and drought stress are important abiotic stresses that have adverse effects on plant growth and productivity. This study summarizes the effects of salt, temperature, and drought stress on growth and *CF* parameters along with chlorophyll and proline content in grafted watermelon seedlings at three true-leaf stages. The results showed the differential effects of each stress on growth performance and the photosynthetic apparatus. The magnitude of the effect was dependent on the type of stress and the duration of seedling exposure to stress. We found a significant decrease in growth parameter values under all stress conditions, which was consistent with previous reports in a range of plants, including watermelon (Hou et al., 2016; Bhandari et al., 2018; Yanyan et al., 2018; Lee et al., 2021; Shin et al., 2021a).

Similar to several previous studies in various plants (Bhandari et al., 2018; Shin et al., 2020a, 2021b), growth parameter values were significantly reduced in all three experiments. However, the magnitude of the variation was dependent on the stressor. There were clear differences between the control and salinity-stressed seedlings. Changes in leaf phenotype and roots of plants subjected to salinity stress and a decrease in fresh shoot weight of scions were previously observed in watermelon, *Arabidopsis*, lettuce, and tomato (Kamanga et al., 2020; Rolly et al., 2020; Shin et al., 2020a; Song et al., 2020). In addition, the poor growth status showing leaf deformation and stunted seedling growth under drought stress conditions was similar to the previous report by Zhang et al. (2011) that might be due to physiological changes in the leaves, nodes, and stems, including decreased chlorophyll content and inhibition of photosynthesis due to lack of water (Zhang et al., 2019). Similar results have been reported for tomato, watermelon, and other plants (Omprakash et al., 2017; Moles et al., 2018; Li et al., 2019). These results were similar to those of previous studies, which found that the chlorophyll content of plants affected by drought stress was reduced in watermelon, tomato, lettuce, and *Arabidopsis* affected by drought stress (Banks et al., 2018; Yao et al., 2018; Malambane et al., 2021; Shin et al., 2021a). Our results also showed a decrease in growth parameters under high and low temperature conditions, which is in accordance with Hou et al. (2016). In addition, high-temperature and low-temperature stress causes damage to the cellular structure of plants, reduction of chlorophyll, and deterioration of photosynthetic function (Garstka et al., 2007; Mattila et al., 2020). The result showed more prominent effect of low temperature compared to the high temperature stress this was because the stock cultivar used in this study was resistant to the high temperature. Phenotypic changes in the leaves and roots of plants subjected to low-temperature stress and a decrease in fresh shoot weight of scions were also observed in *Arabidopsis* and watermelon seedlings (Mattila et al., 2020; Lu et al., 2021).

### Effect of Salt, Temperature, and Drought Stress on *CF* Parameters

*CF* analysis can sensitively detect changes in photosynthetic activities in plants and has been used to study the response of plants to biotic and abiotic stresses (Gorbe and Calatayud, 2012; Shin et al., 2020a,b, 2021a). *CF* has been widely used as a non-destructive evaluation technique to evaluate the photosynthetic level of plants under salinity stress (Morant-Manceau et al., 2004; Bhandari et al., 2018; Shin et al., 2020b). However, the response to different stresses is highly dependent on the magnitude, type, and duration of stress undergone by the plants, and on plant genotypes. In this study, *CF* parameters responded differentially depending on the type of stress, although the general trends of some parameters were similar. Plants exposed to stressful conditions exhibited a decreasing trend in photochemical quenching and an increase in non-photochemical quenching parameters, although non-photochemical quenching parameters also decrease at severe stressful conditions (Murchie and Lawson, 2013).

Fv/Fm, an important photochemical quenching parameter that determines the maximum quantum efficiency under dark conditions, showed a similar value (~0.80) throughout the experimental period under control conditions in all three experiments. These results are consistent with a large number of other studies on unstressed plants (He et al., 2009; Bhandari et al., 2018; Shin et al., 2020b, 2021a). Salt stress induced a decrease in Fv/Fm values, but the decrease was significant only at higher salinity levels after 8 days of treatment. Similar results were also previously found in a range of plants, including watermelon (Hou et al., 2016; Shin et al., 2020b). In accordance with Bhandari et al. (2018), we found a significant decrease in Fv/Fm values in seedlings grown under low temperature, whereas high temperature did not affect Fv/Fm until the end of the experiment, indicating that higher temperatures did not damage the primary photochemical reaction sites; stroma of chloroplasts and thylakoid lamellae which are the primary sites of heat injury (Wise et al., 2004). In the drought stress treatment, we found non-significant changes in Fv/Fm throughout the treatment period. Such differential effects on Fv/Fm by abiotic stresses have also been observed in a range of plants (Fahad et al., 2017; Giordano et al., 2021), however, this is the first report that provides information on Fv/Fm values under three stress factors at the same time.

The other photochemical quenching parameters [Fv′/Fm′, Y(PSII), and qP] were also affected when exposed to stress conditions. All of them exhibited similar trends as in the case of Fv/Fm, although the magnitude was different, showing lower values when exposed to higher salt stress (>50 mM). In the case of temperature stress treatment, we found significantly lower values of Fv′/Fm′ and Y(PSII) from days 2 and 4 of treatment onward, respectively. In contrast, qP showed non-significant changes. Furthermore, only Y(PSII) values exhibited significant differences between control and drought-stressed seedlings, and even in that case, the difference was statistically significant at the end of the experiment. The decrease in the Fv′/Fm′ values in the seedlings grown under lower temperatures might be due to chilling injury caused by cold stress under the given conditions (Hou et al., 2016). All the photo chemical quenching parameters were affected by temperature stress suggesting the disturbed homeostasis *via* the modification in carbon metabolism enzymes, starch accumulation, and sucrose synthesis, by down regulating the genes in carbohydrate metabolism (Ruan et al., 2010).

Values of the non-photochemical quenching parameters NPQ and qN decreased with increasing salinity level and treatment time. In this case, we also found a significant effect at higher salinity levels (>50 mM). The adverse effect was observed at 150- and 200-mM salinity levels. In the second experiment with temperature stress conditions, both the high and low temperatures had non-significant effects on qN when compared to the control at the respective treatment time. In contrast, both high and low temperatures exhibited significant differences in NPQ values at the respective treatment times. NPQ levels generally increase under stressful conditions and decrease under severe stressful conditions (Murchie and Lawson, 2013), and the magnitude of changes depends on the plant species and the level of stress (Yao et al., 2018; Shin et al., 2020a). Seedlings grown under high salinity level and low and high temperatures showed a significant decrease in NPQ levels when compared to control seedlings during the respective treatment time, indicating a reduction in the heat dissipation capacity, limitations of CO_2_ assimilation, and imbalance of photochemical activity in photosystem II (Huang et al., 2019). In addition, our results also indicated the incapacity of the protection mechanism due to senescence for the downregulation during those periods (Ors et al., 2021). Furthermore, non-significant changes in NPQ levels until the end of the experiment under drought conditions indicated that the photochemical activity in the photosystem was not severely influenced until that period.

**Figure 11:**
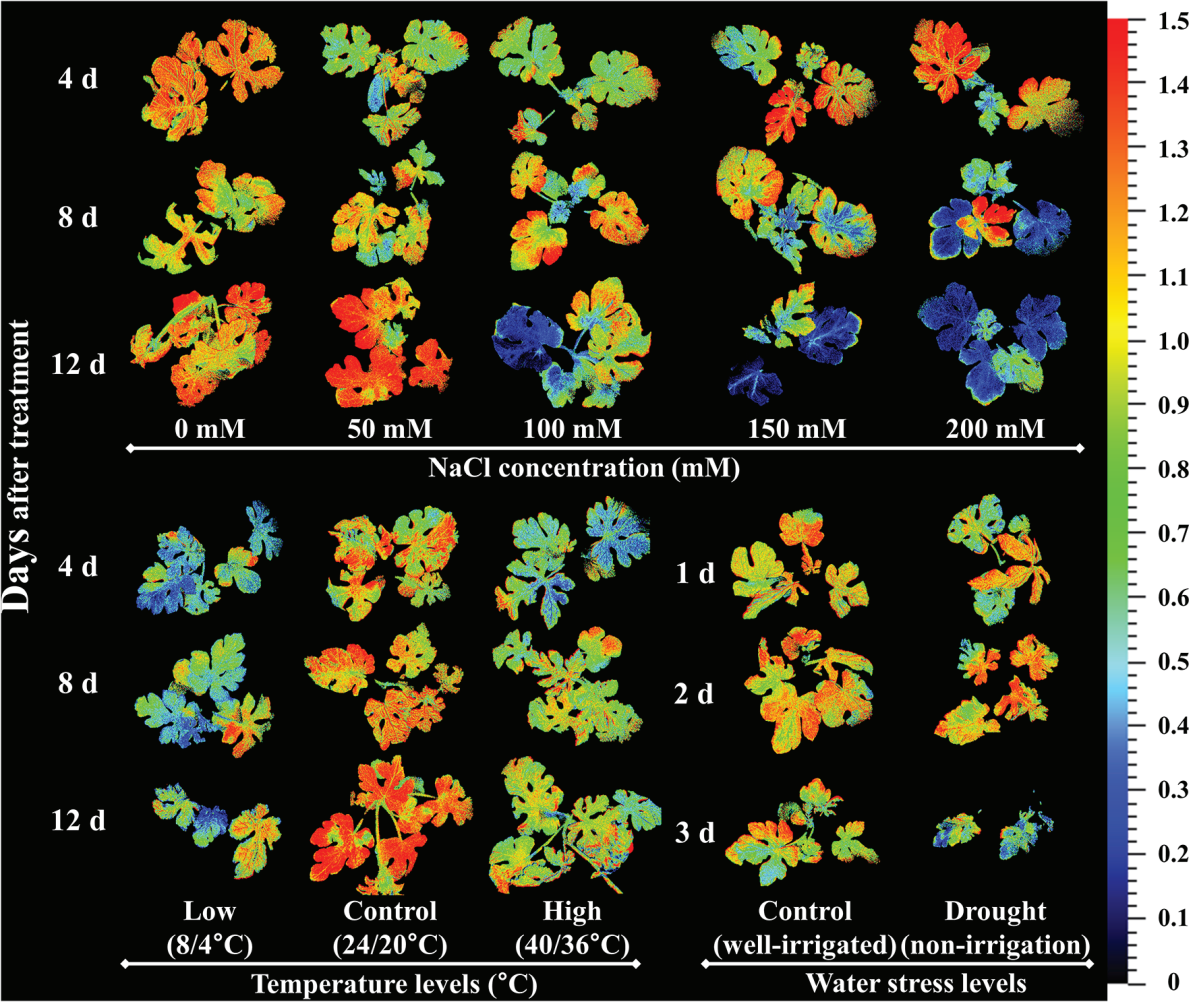
Representative chlorophyll fluorescence images of non-photochemical quenching (NPQ) under the salinity, drought, and temperature stress treatment in different time after each stress initiation in watermelon seedlings.

Y(NO) is an important *CF* parameter that indicates the effectives of the photo-protective mechanisms (NPQ) in plants. The increase in Y(NO) levels at a higher salinity levels and at lower temperature conditions implied that the seedlings received extreme stress during the experiment, and the NPQ was decreased under extreme stress conditions (Murchie and Lawson, 2013; Huang et al., 2019). These results are consistent with previous reports on watermelon and tomato seedlings (Hou et al., 2016; Shin et al., 2020a). Similar to the effects on NPQ, Y(NO) levels also exhibited non-significant changes until the 2^nd^ day of treatment time in drought stress treatment, implying that the photosynthetic activity was normal until that period. Rfd, an indicator of plant vitality under stressful conditions (Murchie and Lawson, 2013), showed a significant decrease in high salinity levels from day 4 of the experiment on salinity stress experiment. Our results were consistent with those of Shin et al. (2020b), who also found a significant decrease in Rfd levels in tomato seedlings exposed to extreme salinity stress. Both the high-and low-temperature stress also showed a significant decrease in Rfd levels from the beginning of the experiment; however, low temperature had a more prominent effect on Rfd levels, in accordance with Hou et al. (2016). Drought stress had minimal effect only at the end of the experiment, suggesting that the photosynthetic protective mechanism of watermelon seedlings is affected only under extreme drought conditions (soil water level < 40%). NPQ, which was the common parameter significantly affected by each of the stressor, is presented as a representative chlorophyll fluorescence images to understand the spatial heterogeneity (Figure 11). The results showed the obvious spatial variation in *CF* images but differently depending upon the stressor.

The response of *CF* parameters to salt, temperature, and drought stress conditions were different from each other. Fv/Fm, NPQ, Rfd, and Y(NO) showed significant changes during the progressive treatment time at higher salinity levels, suggesting the possible use of these parameters to detect salinity stress. Only two *CF* parameters; Rfd and NPQ were significantly affected under high temperature conditions even the cultivar was high-temperature resistant, while six parameters [Fv/Fm, Fv′/Fm′, Rfd, Y(NO), Y(PSII), and NPQ] were affected significantly under low temperature conditions. So, two parameters (Rfd and NPQ) can be used to detect low-and high-temperature stress, as these parameters decreased significantly from the beginning of the experiment under both temperature conditions. Furthermore, Rfd and NPQ levels decreased significantly at the end of the experiment in the drought stress treatment and could be considered for detecting drought stress. Further studies on seedlings of many watermelon genotypes exposed to long-term stress and higher light conditions might be required to detect the effect of genotype, and the impact of initial severe stress as genotype is also a key factor for the tolerance of stress conditions.

### Effect of Salt, Temperature, and Drought Stress on Chlorophyll and Proline Content

Proline, one of the osmoprotectants (glycine, proline, betaine, and soluble sugars) is an important compatible solute found in plants (Farooq et al., 2008). It is generally elevated in large amounts in plants under stressful conditions. It is found in plants in small quantities even under non-stressed conditions (Trovato et al., 2008; Gupta et al., 2014). It helps to maintain membrane integrity by maintaining turgor pressure to sustain the growth of the plant (Gupta et al., 2014). The level of proline content is highly dependent on plant genotypes and stressors (Nikolaeva et al., 2010). Proline increased with the increase in stress level in all three experiments compared to the respective control seedlings, and the magnitude of increment was dependent on the stressor. We found a significant increase in proline content with increasing salinity and progressive treatment time. The highest proline levels were found in the seedlings treated with the highest salinity level, similar to previous reports on lettuce, tomato, and citrus (Shin et al., 2020a,b; Martínez-Cuenca et al., 2021). The elevation of proline levels at different salinity levels indicated that seedlings grown at the salinity level experienced high stress levels. Our results showed that the seedlings grown under both high and low temperatures had statistically higher proline content than the control at both 6 and 12 days of treatment. However, the highest proline content was observed at high temperatures, which is consistent with previous results in paprika seedlings (Bhandari et al., 2018). The over accumulation of proline under high temperature stress was responsible to regulate the osmotic activities and protect cellular structure by maintain the membrane stability and by buffering the cellular redox potential (Farooq et al., 2008). So the status of the plants was maintained with normal photosynthesis showing non-significant changes in Fv/Fm. Similarly, seedlings experiencing drought stress also showed statistically higher proline content; however, the magnitude of increase was lower than that in previous reports (Li et al., 2019; Shin et al., 2021a), which might be due to the difference in genotypes and less treatment time. These results suggest that a responsive mechanism exists due to salt, temperature, and drought stress in grafted watermelon seedlings, which along with other compounds also helps to generate an efficient antioxidant system to cope with ROS species, increase the protein stability, and stabilize the structure of the membrane bilayer (Mirzaei et al., 2012). Comparative analysis showed that the highest proline accumulation and its fluctuation occurred under temperature stress, followed by salinity stress and drought stress.

Chlorophylls are photosynthetic pigments that are responsible for the photosynthetic efficiency of plants and are ultimately responsible for primary production (Gitelson et al., 2003). Chlorophyll a and b levels were measured in all three experiments, and we found significant changes in the levels of both chlorophylls with increasing stress levels. The levels of both chlorophyll a and b decreased with increasing salinity levels during the progressive treatment time, consistent with previous reports in different vegetables, including watermelon (Taïbi et al., 2016; Yanyan et al., 2018; Shin et al., 2020a, 2021a). The lower content levels of the chlorophylls at higher salinity levels was probably due to damage to the chloroplast membrane and structure, increased activity of chlorophyllase, and photo-oxidation of chlorophyll due to the excessive accumulation of salt in the soil (Taffouo et al., 2010; Silveira and Carvalho, 2016). Temperature stress treatment showed a somewhat different accumulation pattern of chlorophylls, and the effect was lower than that of salinity stress. As watermelon is a thermophilic crop, it is less affected at high temperatures than at low temperatures, and seedlings grown at low temperatures exhibited relatively higher chlorophyll content than those grown at high temperatures. Our results were consistent with previous reports by Hou et al. (2016), who also found lower chlorophyll content in watermelon seedlings grown at cold temperatures. This is because plants exposed to low temperatures experience chilling injury, which enhances ion imbalance, reduction in antioxidant activity, and low chlorophyll content (Lu et al., 2020; Mlinarić et al., 2021). Chlorophyll content in drought-stressed seedlings was least affected among the three treatment types, although significant changes were observed between control and drought-stressed seedlings during the respective treatment schedule. In the drought stress experiment, the treatment was conducted for only 3 days as the status of the seedling was poor after that period. Previous reports also showed that chlorophyll levels in plants decrease after severe stress; however, some authors have reported an increase in chlorophyll content for some time and decrement after exposure to severe stress conditions (Partelli et al., 2009; Bhandari et al., 2018; Shin et al., 2020b, 2021a).

## Conclusion

This study showed the potential of using *CF* imaging to detect abiotic stressors (salinity, high and low temperature, and drought) in grafted watermelon seedlings (Figure 11). The response of the seedlings to various abiotic stresses was observed through changes in *CF* and growth parameters, chlorophyll, and proline content. The changes were dependent on the type of stressor and duration. Most *CF* parameters were affected only at the higher salinity stress (>50 mM), with the most influential parameters being Fv/Fm, NPQ, Rfd, and Y(NO) increased during the progressive treatment time. Low temperature had a prominent effect on nearly all the *CF* parameters compared to the high temperature stress, suggesting that the low temperature caused more severe photo-inhibition of photosynthesis than high temperature. Drought stress had a similar effect on *CF* parameters as in the high-temperature stress, showing significant changes only in the Rfd and NPQ. Altogether, NPQ and Rfd could be used as index parameters for the detection of three abiotic stresses. In general, values of all the growth parameters reduced, chlorophyll content levels were decreased or increased depending upon the stressor, and proline content was increased in the seedlings exposed to each stressor. These results imply that photosynthetic activity, growth performance, and chlorophyll and proline content are differentially affected by each stressor and their magnitude, which might be useful for the effective detection of each stress during the production process of watermelon-grafted seedlings. Furthermore, research on open environmental conditions having high light condition and combined application of stressors are required for understanding the comparative and synergetic effects of these stressors, respectively in photosynthetic and growth parameters in watermelon seedlings.

## Data Availability Statement

The original contributions presented in the study are included in the article/supplementary material, further inquiries can be directed to the corresponding authors.

## Author Contributions

YKS designed and performed the experiments, statistically analyzed and interpreted the data, and wrote the manuscript. SRB designed the experiment, analyzed and interpreted the data, and wrote the manuscript. JGL conceived the project, designed the experiment, and wrote the manuscript. All authors contributed to the article and approved the submitted version.

## Funding

This research was funded by the Basic Science Research Program through the National Research Foundation of Korea (NRF) by the Ministry of Education (no. 2019R1A6A1A09031717).

## Conflict of Interest

The authors declare that the research was conducted in the absence of any commercial or financial relationships that could be construed as a potential conflict of interest.

## Publisher’s Note

All claims expressed in this article are solely those of the authors and do not necessarily represent those of their affiliated organizations, or those of the publisher, the editors and the reviewers. Any product that may be evaluated in this article, or claim that may be made by its manufacturer, is not guaranteed or endorsed by the publisher.
